# Automatic inhibition of habitual response associated with a non-target object while performing goal-directed actions

**DOI:** 10.1177/1747021820971921

**Published:** 2020-11-21

**Authors:** Lari Vainio

**Affiliations:** 1 Helsinki Collegium for Advanced Studies, University of Helsinki, Helsinki, Finland; 2 Perception, Action & Cognition Research Group, Department of Psychology and Logopedics, Faculty of Medicine, University of Helsinki, Helsinki, Finland; 3 Phonetics and Speech Synthesis Research Group, Department of Digital Humanities, University of Helsinki, Helsinki, Finland

**Keywords:** Habitual response, affordance, response inhibition, behaviour control

## Abstract

This study is devoted to investigating mechanisms that inhibit habituated response associated with affordance of a non-target while executing action directed to a target. In four experiments, a paradigm was used that required a rapid left- or right-hand response according to the direction of the target arrow presented simultaneously or in close temporal proximity to a non-target whose handle position afforded grasping with the left or right hand. In general, responding was decelerated and more erroneous when the handle position was compatible with the responding hand. This effect of response inhibition was removed when the delay between the non-target offset and target onset was longer than 200 ms, and reversed into response facilitation when the target onset was delayed for 400–600 ms. The study suggests that processes that control withholding habitual response associated with affordance of a non-target utilise response inhibition mechanisms overlapping with those involved in behavioural control of the stop-signal task. This response inhibition is triggered automatically and directly by affordance of a non-target without preceding response excitation associated with this affordance cue.

## Introduction

People develop various action habits during their lifetime such as turning on the faucet or flipping the light switch. Habits can be considered as actions automatically (i.e., with minimal or no conscious and effortful top-down control of action planning) elicited by the environmental stimuli via direct stimulus–response (S-R) links that are developed through repetition and learning (Robbins & Costa, 2017). Of course, this does not mean that people could not withhold executing actions associated with these habituated stimuli if they do not benefit the current behavioural goal (Hardwick et al., 2019). Without the ability to inhibit habitual actions, we would be impulsive creatures that could not optimally execute goal-directed behaviour. Hence, to understand human behaviour, it is particularly important to understand the mechanisms that enable executing goal-directed behaviour while withholding responding to habituated stimuli that are irrelevant to the current goal. However, processes related to controlling habitual behaviour are far from understood.

In one type of paradigm exploring habitual responses in humans, participants acquire habitual associations between artificial S-R mappings (e.g., press the key with the middle finger when you see the letter L and with the index finger when you see the letter K) through intensive practice that can last for several days (e.g., Hélie et al., 2010). However, ecological validity of this kind of method in researching habitual responses is relatively weak. Importantly, if complying with the above-mentioned definition of habitual actions, it is noteworthy that research exploring habitual responses has also focused on S-R associations that are generated via long-term naturalistic bodily interaction with objects (e.g., Tucker & Ellis, 1998, 2001). Affordance is a concept that often provides a theoretical basis for this research tradition. Gibson (1979) originally introduced the affordances referring to visual cues of an environment that automatically establish for an individual what actions are possible. In line with this proposal, considerable evidence has shown that viewing objects containing functionally meaningful physical characteristics can automatically potentiate corresponding actions (e.g., Franca et al., 2012).

A handle can be assumed to provide the most typical object affordance to which people are habituated to respond in a specific manner. As an example, when one approaches a closed door, the door handle is automatically, with minimal or no conscious effort, reached for and grasped by the hand that is the most suitable for manipulating the handle. Indeed, various behavioural (Tucker & Ellis, 1998), brain stimulation (Buccino et al., 2009), and electrophysiological (Goslin et al., 2012) studies have shown that a handle affordance can automatically bias response selection by evoking the action representation of the hand which is the most suitable for grasping the handle.

However, an environment often presents us with more than one object towards which action could be directed. Given that actions can be, however, directed towards one graspable object at a time, it is important that action is guided by information from a single, selected target object, while escaping the competitive information from habituated properties of non-selected objects, at least when the objects are not requesting for a integrated behavioural goal (Xu et al., 2015). Many accounts of sensorimotor control assume that a non-target’s representations, including action representations, are associated with inhibition during selections of the target (Houghton & Tipper, 1994). This article investigates inhibitory mechanisms that enable withholding execution of response, which is habituated to handle affordance of a non-target, while performing goal-directed actions towards the target object. These mechanisms are explored by recruiting the negative handle affordance (NHA) paradigm originally introduced by Vainio et al. (2014, 2011). In particular, the study investigates whether the NHA effect could be based on the same inhibitory control processes as other negative priming effects, and whether the NHA effect is based on automatic inhibition control processes or whether voluntary inhibition of unwanted response has some role in the effect. However, before introducing the research questions and the paradigm used in the study in detail, the article discusses previous research showing how affordance characteristics of an object can automatically activate matching actions and how it seems that actions associated with affordance of a non-target object are inhibited.

### Object affordances and action potentiation

Separate visual streams process objects for perceptual and behavioural purposes (Milner & Goodale, 1995). The ventral stream, which recruits mostly the temporal cortex, is largely responsible for categorization and identification of objects. The dorsal stream, including certain parietal and premotor areas, mostly processes action-relevant properties of objects for behaviour guidance (Borra et al., 2017; Murata et al., 2000; Raos et al., 2004, 2006). One of the core functions of the dorsal stream is to automatically transmit orientation information of an object to guide selection of the responding hand and rotation of the selected hand so that the object would be grasped most effectively (Arbib, 1981). Indeed, in monkeys, some of the anterior intraparietal (AIP) neurons code the orientation of visual objects for transmitting this information into motor programmes (Fagg & Arbib, 1998; Murata et al., 2000).

It has been proposed that the dorsal stream can be segregated into two functionally separate streams (Binkofski & Buxbaum, 2013; Borghi & Riggio, 2009). The dorso-dorsal stream, which employs superior parietal areas, guides action planning based on currently visible structural and spatial properties of objects (e.g., size, shape, and orientation of axis of elongation), while the ventro-dorsal stream, which employs the inferior parietal areas, guides action planning based on functional object properties such as learned semantic knowledge about a handle. In line with the view that a functional handle can automatically activate corresponding grasp representations, solely viewing objects whose functional handle is pointing towards the right hand increases corticospinal excitability (CSE) in the grasp-related muscle of the right hand, that is, the first dorsal interosseous (FDI) muscle (Buccino et al., 2009). Similarly, Bolton et al. (2019) found increased CSE in the FDI when participants passively viewed a wall-mounted safety handle compared with conditions where the handle was covered and therefore not visible.

The most commonly used method that has investigated how handle affordances influence selecting the hand of response was originally introduced by Tucker and Ellis (1998). In that paradigm, participants are typically presented with graspable household objects (e.g., a hammer) with handles oriented towards the left or right hand. Participants can be asked to respond with the left or right hand to indicate, for example, whether the object presented belongs in either a kitchen or a toolbox category (McBride et al., 2012a). In this kind of task, responses are typically performed faster and more accurately when the handle position is compatible with the responding hand. This effect has been often explained in the same way as the findings of the above-discussed transcranial magnetic stimulation (TMS) studies according to which handle affordance automatically potentiates grasping behaviour of the hand that is congruent with the handle position (Ellis, 2018).

### Non-target-related object affordances and response inhibition

Pavese and Buxbaum (2002) used a version of the flanker paradigm (Eriksen & Eriksen, 1974) to show that when the handle affordance information is associated with a non-target object, the handle position slows down responses executed with the hand compatible with the handle of this non-target. Their participants were required to reach to grasp or reach for the target (i.e., blue mug) presented next to a non-target (i.e., purple mug) with the right hand. The responses were performed significantly slower when the handle of the non-target was pointing towards the responding hand in comparison with the non-responding hand. Later, Ellis et al. (2007) presented similar negative compatibility effect relative to the size affordance of a non-target. That is, precision and power grip responses were inhibited when the size affordance of the non-target was compatible with the required grip. According to Caligiore et al. (2013), these non-target-related negative compatibility effects were observed because the non-target representations, including action representations, are inhibited when selecting the target for responding.

Most relevantly for the current study, Vainio et al. (2011, 2014) observed a corresponding negative compatibility effect relative to handle affordance of a non-target by using a priming paradigm. In that task, the participants were briefly (30 ms) presented with a non-target mug 50 ms before the onset of the target arrow, which was presented at the same spatial location as the centre of the main body of the prime mug. A handle of the mug was oriented towards the left or right hand. The participants were required to respond, as fast as possible, according to the pointing direction of the target arrow. As predicted, responding was significantly slower and more inaccurate when the response was performed with the hand that was compatible with the handle of the prime mug (the negative handle affordance, NHA, effect). This suggests that the response associated with the prime handle is inhibited while carrying out the response to the target. Moreover, in the control condition, the prime mug was replaced by the unfamiliar object whose handle component was not different from the mug. However, critically vertical spatial features of the mug-like object were manipulated so that the participants did not recognise the object as a mug. Contrary to the mug stimuli, these mug-like objects produced a positive priming effect in which responses were facilitated when the handle position was compatible with the responding hand. Vainio et al. (2014) presented electrophysiological evidence for the inhibition account of the NHA effect.

### Research question on whether the NHA effect is based on similar mechanisms as other negative priming effects

The present study explores whether the NHA effect could be based on the same inhibitory control processes as other negative priming effects. Typically, priming effects present decelerated response speed when there is incompatibility between the non-target and the response (e.g., Becker, 1980; Taylor, 1977). As an example, semantic priming and repetition priming present common paradigms to explore priming mechanisms, and most typically result in positive priming effects. One of the differences between semantic priming and repetition priming concerns the longevity of the effects: Repetition priming may persist even for hours, while semantic priming is typically dissipating over the course of several seconds (Wagner & Koutstaal, 2002). However, regardless of these differences, all major priming phenomena have been proposed to be mostly based on S-R bindings (Henson et al., 2014).

Importantly, the negative compatibility effects associated with the affordance of a non-target are not the only behavioural effects that demonstrate delayed responding when the required response is compatible with some perceptual aspect of the non-target prime object. Various types of negative priming effect (Eimer & Schlaghecken, 1998; Tipper, 1985) illustrate similar response inhibition related to an ignored non-target object. It has been proposed that these negative priming effects are – similarly to other major priming effects – based on SR binding mechanism, and at least partially linked to the response inhibition mechanisms (Bowman et al., 2006; Houghton & Tipper, 1994). Therefore, as already proposed by Vainio et al. (2011, 2014), it is justified to assume that the NHA effect could be based on response control mechanisms somewhat similar to these negative priming effects.

These negative priming effects can be observed if the non-target is effectively ignored and/or presented subliminally. In the traditional negative priming task (e.g., Tipper, 1985), negative priming was explored using a prime–probe paradigm. In this task, participants are required to respond to a probe object that is presented after the prime stimuli, which is constructed from two overlapping objects (e.g., a red cat and a green car). In addition to responding to the probe, participants have to ignore one of the primes (e.g., a red cat) and pay attention to the other prime as it has to be recalled later. In this task, participants are slowed in responding to the identity of a target that has just served as an ignored prime. More recent studies have shown that similar negative priming can be observed with a single subliminally presented prime (Frings & Eder, 2009; Frings & Wentura, 2005).

In one version of negative priming, participants are presented with a subliminal prime arrow (i.e., it is presented for 20 ms and is backward-masked) pointing to the left or right prior to presenting a left or right pointing target arrow. They are required to respond with the right or left hand based on the direction of the target arrow. Eimer and Schlaghecken (1998) originally showed that in this task, responses are made slower if the direction of the prime arrow is compatible with the direction of the target arrow.

The theoretical models that explain negative priming associated with a non-target object typically assume that these effects are caused by mechanisms of sensorimotor control that direct inhibitory processes towards the non-target-related response activation (e.g., Bowman et al., 2006; Caligiore et al., 2013; Houghton & Tipper, 1994). That is, one of the primary principles of the models constructed to explain negative priming assumes that the prime initially evokes a short-lived response excitation related to the response compatible with the identity of the prime because also a non-target can induce response activation prior to selecting the target object. Regarding subliminal arrow priming, the lateralized readiness potential (LRP) pattern shows this initial response activation of motor processes relative to the response-compatible prime, which occurs before the onset of the inhibitory reversal phase (Eimer & Schlaghecken, 1998). This initial prime-induced response facilitation is also observed in behavioural response patterns. Positive arrow priming can be observed when the interstimulus interval (ISI) between the offset of the backward mask and the onset of the target arrow is substantially short (i.e., 0 or 32 ms), and when reaction times are analysed in deciles showing advantages for compatible trials in very fast responses (Eimer, 1999).

Furthermore, Bowman et al.’s (2006) model assumes that with an adequately long ISI between the prime and target, there is sufficient time for the inhibitory reversal to complete and for activation to reverse again. This phenomenon has been observed in behavioural effects showing a strong negative arrow priming at an interval of 150 ms between backward-mask onset and target onset, followed by a weak but significant positive arrow priming effect at an interval of approximately 300–500 ms (Lingnau & Vorberg, 2005; Sumner & Brandwood, 2008). Regarding the negative priming linked to the prime-probe task, the time course of the effect is more long-lasting than that of the subliminal arrow priming. In a between-subjects design, inhibition of negative priming can be observed in responses even when the delay between the prime and probe is up to 7 s (Tipper et al., 1991). When varying delay intervals are introduced in a within-subjects design, the negative priming persists for intervals of up to 2500 ms (Hasher et al., 1996).

In the original paradigm of the NHA effect, there was a 50 ms delay between the offset of the prime and the onset of the target. If the NHA effect is based on the same inhibitory processes as negative priming and/or subliminal arrow priming, we might be able to observe a positive compatibility effect between the handle position of a prime and the responding hand by minimising the delay between the prime and target. This research question is investigated in Experiment 2. Experiments 3–4 further explore the time course of the NHA effect by delaying the ISI between the offset of the prime and onset of the target. If the NHA effect is based on inhibitory mechanisms similar to the subliminal arrow priming, the negative compatibility effect should decay rapidly (e.g., within 200 ms) after offset of the prime and reverse into a positive compatibility effect in longer ISI conditions (e.g., 400 ms). In contrast, if the NHA effect is based on response inhibition mechanisms similar to the negative priming linked to the prime-probe task, the effect should be observed even in the longest delay conditions.

### Research question on whether the NHA effect is based on automatic or voluntary inhibitory control of behaviour

The current study also investigates whether the NHA effect is based on automatic inhibition control processes or whether voluntary inhibition of unwanted response has some role in the effect. Present view is that overlapping processes are responsible for automatic and voluntary response control (McBride et al., 2012b; Sumner & Husain, 2008; Verbruggen et al., 2014). However, traditionally the inhibition of a motor response has been conceptualised as a voluntary act of cognitive control (e.g., Logan, 1983; Logan & Cowan, 1984). This is particularly the case in the research paradigms that use the stop-signal or go/no-go paradigms (Logan & Cowan, 1984; Verbruggen et al., 2014; Verbruggen & Logan, 2008) in which participants have to voluntarily stop the initiated response when the stop signal is detected or withhold responding in the no-go trials. In contrast, negative priming effects have to mostly operate within automatic inhibition processes because they are only observed if the prime is presented subliminally. However, the NHA effect is observed with supraliminally presented prime objects, opening the possibility that it would be caused by voluntary response control processes.

Indeed, Liu et al. (2016) proposed that the NHA effect is based on a voluntary strategy of intentionally avoiding responding to the target arrow with the hand that is compatible with the handle position of the prime. It was proposed that the critical aspect of this effect is that Vainio et al. (2011, 2014) instructed their participants to pay as little attention as possible to the prime mug. In line with this view, it has been shown that instructing participants to intentionally ignore the prime can be a critical aspect in negative priming effects (Ortells et al., 2003). To test this assumption, the present study explores whether the NHA effect can be observed when participants are only instructed to respond to the arrow without saying anything about the prime—not even that the arrow is preceded by the appearance of a prime. Consequently, in all of the four experiments of the present study, the appearance of the prime prior to target onset was not instructed to the participants. Second, Experiments 1 and 2 investigated this question of influence of voluntary strategy on the effect by explicitly asking participants whether they used some intentional strategy while performing the task to perform the task as rapidly and accurately as possible. Furthermore, given that supraliminally presented prime can induce response effects that are based on strategic processes in relatively long prime-target intervals, while automatic priming can occur when prime-target intervals are significantly shortened (Jaśkowski, 2008), Experiment 2 explores this question of voluntary strategy by presenting the prime and target at the same time. One might assume that when the prime and target are presented briefly (for 50 ms) at the same time rather than presenting the prime before the target, which is the case in the standard NHA paradigm, it would be more difficult to use the prime as some sort of strategic cue according to which the target-related responses would be adjusted. Consequently, one might expect that in this stimulus condition the effect would not be observed if the effect is based on this kind of voluntary strategy.

## Experiment 1

The task used in Experiment 1 was the one that has been previously used to explore the NHA effect (Liu et al., 2016; Vainio et al., 2011, 2014) with the exception that the participants were not instructed about the appearance of the prime. The other difference compared with the previous studies was that Experiment 1 used a jug as a prime stimulus, whereas the previous studies used a mug as a prime. However, at this point, it is important to highlight that some researchers have challenged the affordance explanation of the handle affordance effects originally presented by Tucker and Ellis (1998) by proposing that the effects are based on visuomotor mechanisms that are mostly inseparable from the mechanisms underlying the Simon effect (Simon, 1969). According to these views, the handle affordance effect is based on processing the location of the most salient object part, or the location of the object part that is the most relevant to the experimental task relative to the left–right response setup (Cho & Proctor, 2010; Proctor et al., 2017). The previous NHA study verified that the effect reflects a genuine handle affordance effect by comparing the influence of a mug prime and mug-like prime on behavioural and electrophysiological responses (Vainio et al., 2011, 2014). To verify the applicability of a jug prime in investigating a genuine handle affordance effect, in Experiment 1 the jug prime is presented in either an upright or inverted position. Riddoch et al. (1998) have shown that the handle information of the reached mug loses its capacity to evoke responses when it is not recognised as a familiar graspable object or when the functional meaning of the mug is diminished by inverting it. Based on observations of Riddoch et al. (1998), it was expected that if the NHA effect were observed with the upright jug instead of the inverted jug, this would suggest that the effect is triggered by functional handle affordance information rather than some abstract, spatial cues of the stimuli.

Furthermore, in the subliminal arrow priming, the negative compatibility effect does not disappear with longer reaction times (Seiss et al., 2014). In contrast, the fastest reaction times are associated with a positive compatibility effect (Eimer, 1999). Given that one of the primary aims of the study was to investigate whether the NHA effect is based on behavioural control mechanisms similar to the subliminal arrow priming, distributional analysis was also carried out for the data of Experiment 1 so that the reaction time data were divided into four bins based on speed of reaction times.

### Method

#### Participants

Fifteen participants (10 females; 19–47 years of age; mean age = 30.4 years; 2 left-handed) had normal or corrected-to-normal vision. All persons gave their informed consent prior to their inclusion in the study. The selection of number of participants was based on the previous identical investigation of the NHA effect (Vainio et al., 2014), which showed highly significant compatibility effects using 15 (Experiment 1) participants. Our sample size calculations, carried out by using G*power software, were based on the results of that study in which the effect size ($\eta_{p}^{2}$) of the main effect of Compatibility was 0.63 and the power was 0.92. Based on these calculations, the estimated sample size was 15. The study was approved by the Ethical Review Board in Humanities and Social and Behavioural Sciences at the University of Helsinki.

#### Apparatus, stimuli, and procedure

**Figure 1.**
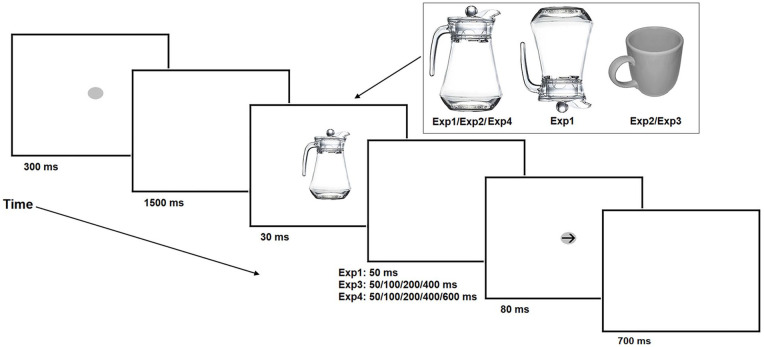
Schematic depiction of the temporal structure of Experiments 1, 3, and 4.

Each participant sat in a dimly lit room with his or her head 70 cm from a 19″ CRT monitor (screen refresh rate: 85 Hz; screen resolution: 1280 × 1024) and with the index finger of each hand resting on two response buttons 30 cm apart and 40 cm in front of the screen. The study consisted of two blocks. There was a short break between the blocks. In both blocks, the prime stimuli consisted of grey-scale images of a jug (see Figure 1) whose handle was pointing towards the left or right hand (subtended a visual angle of 10.6° vertically and 6.8° horizontally). The left-orientation stimuli were created by flipping the right-orientation images horizontally. In one block the jug was presented upright, and in another block it was presented upside down. All participants performed the inverted block before the upright block. That was because the primary point of the inverted block was to explore whether the handle component of the object modulates responses even when it is not processed as part of an object that could be optimally grasped to functionally use it. It was presumed that if the participants were to perform the upright block before the inverted block, this might strengthen linking the handle component to the corresponding response and as such provoke processing the handle information of even inverted jugs as a handle affordance. Indeed, previously learned inhibitory S-R associations linked to a distractor can be transmitted and generalised to performance in a subsequent experimental block (Verbruggen & Logan, 2008). A left/right pointing arrow (0.6° × 1°) was used as a target stimulus. The black arrow was superimposed over a grey disc (1° × 1.3°). All stimuli were displayed on a white background at the centre of the screen (i.e., in both blocks the same centre point of the main body of the jug was in the centre of the screen).

**Figure 2.**
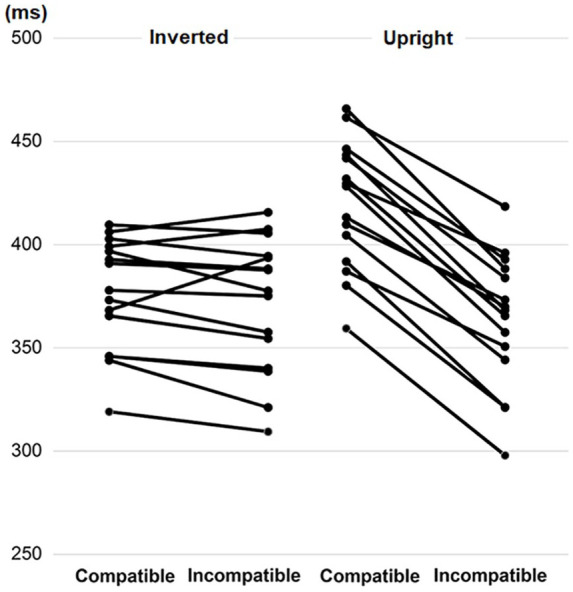
Compatibility effects of Experiment 1 presented for each participant in the inverted and upright block. The figure shows that the negative handle affordance effect is a very robust phenomenon, which can be clearly observed for each participant in the upright block.

Each stimulus was displayed 30 times in each of the four conditions in both blocks. In total, the experiment consisted of 240 trials (30 × 2 [orientation] × 2 [hand of response] × 2 [block]). Each trial started with the presentation of a fixation point that was a grey disc—the same disc as that used as the background of the target arrow. The disc was displayed for 300 ms. Then the disc was replaced by an empty white screen displayed for 1500 ms. Next the prime stimulus appeared on the screen for 30 ms. Then the prime was displaced by an empty white screen for 50 ms. Finally, the target arrow appeared on the screen for 80 ms. The left and right arrows as well as the left and right oriented prime objects were presented in random order with equal probability. A blank white screen was displayed until the participant responded or the trial timed out 700 ms after arrow offset. This time constraint was included in the design to increase the pressure to perform as fast a response as possible given that habituated responses have been shown to influence responding when participants are forced to respond particularly rapidly (~300–600 ms) (Hardwick et al., 2019). The participants were instructed to respond with their right hand if the arrow was pointing to the right and with their left hand if it was pointing to the left. The participants were instructed to respond as quickly as possible while maintaining accuracy. The prime object was not mentioned in the instructions. The experiment began with approximately 20 practice trials. These practice trials were only provided before the block 1 and included only inverted jugs. Figure 2 provides a representation of the trial structure of the experiment.

After the second block, first (1), the participants were asked orally (oral questionnaire) whether they noticed the object that appeared before the arrow, and if they did notice the object, what the object in the first and second block was. Second (2), they were asked whether they noticed that something changed between the trials, and if they did notice that something changed, what it was that changed. Third (3), they were asked whether they felt some untypical or notable experiences during the study. Fourth (4), they were asked whether they used some intentional strategy while performing the task to perform the task as rapidly and accurately as possible. Fifth (5), they were asked what they thought was investigated in the study. Finally (6), they were asked whether they tried intentionally to ignore the prime object so that they could respond to the arrow accurately and whether they tried to avoid responding with the hand that was compatible with the prime orientation. The experimenter wrote down the answers of the participants.

### Results

Reaction times were measured from the onset of the target arrow to the onset of the key press. Errors (i.e., the participant responded with the wrong hand [2.7%] or did not produce any response [0.2%]) were excluded from the reaction time analysis. In addition, reaction times faster than 200 ms (0.13%) were excluded from the reaction time analysis. The cutoff level of 200 ms was selected because it has been shown that responding to a visually presented target takes a minimum of 200–300 ms (Welford, 1980). Hence, it was assumed that responses faster than that were anticipations. The statistical significance of reaction time values (see Supplementary material for the data) was tested by using a random intercept model (linear mixed model) that treated Block (inverted, upright) and Compatibility between prime orientation and arrow direction (compatible, incompatible) as fixed within factors and Subject as random intercept. Selection of error covariance structure was based on Schwarz’s Bayesian information criterion (BIC). Post hoc comparisons were carried out by using the Bonferroni correction. The analysis was carried out using SPSS software package (version 25).

#### Standard analysis of reaction times and errors

After estimating the best fitting error covariance structure (BIC = 37403.32), the analysis of reaction times revealed significant main effects of Compatibility, *F*(1,14) = 148.07, *p* < .001, and Block, *F*(1,14) = 33.74, *p* < .001, as well as interaction between Compatibility and Block, *F*(1,3447) = 227.79, *p* < .001. The pairwise comparisons test showed that in Block 1 (inverted), there was no significant difference between compatible (*M* = 376 ms) and incompatible (*M* = 371 ms) conditions (*p* = .129). However, in Block 2 (upright) responses were performed slower in the compatible (*M* = 420 ms) than incompatible (*M* = 363 ms) conditions, *p* < .001; *d* (Cohen’s *d*) = 1.9. That is, participants preferred to respond with the hand that was incompatible with the handle position. Figures 2 and 4 present this compatibility effect.

**Figure 3.**
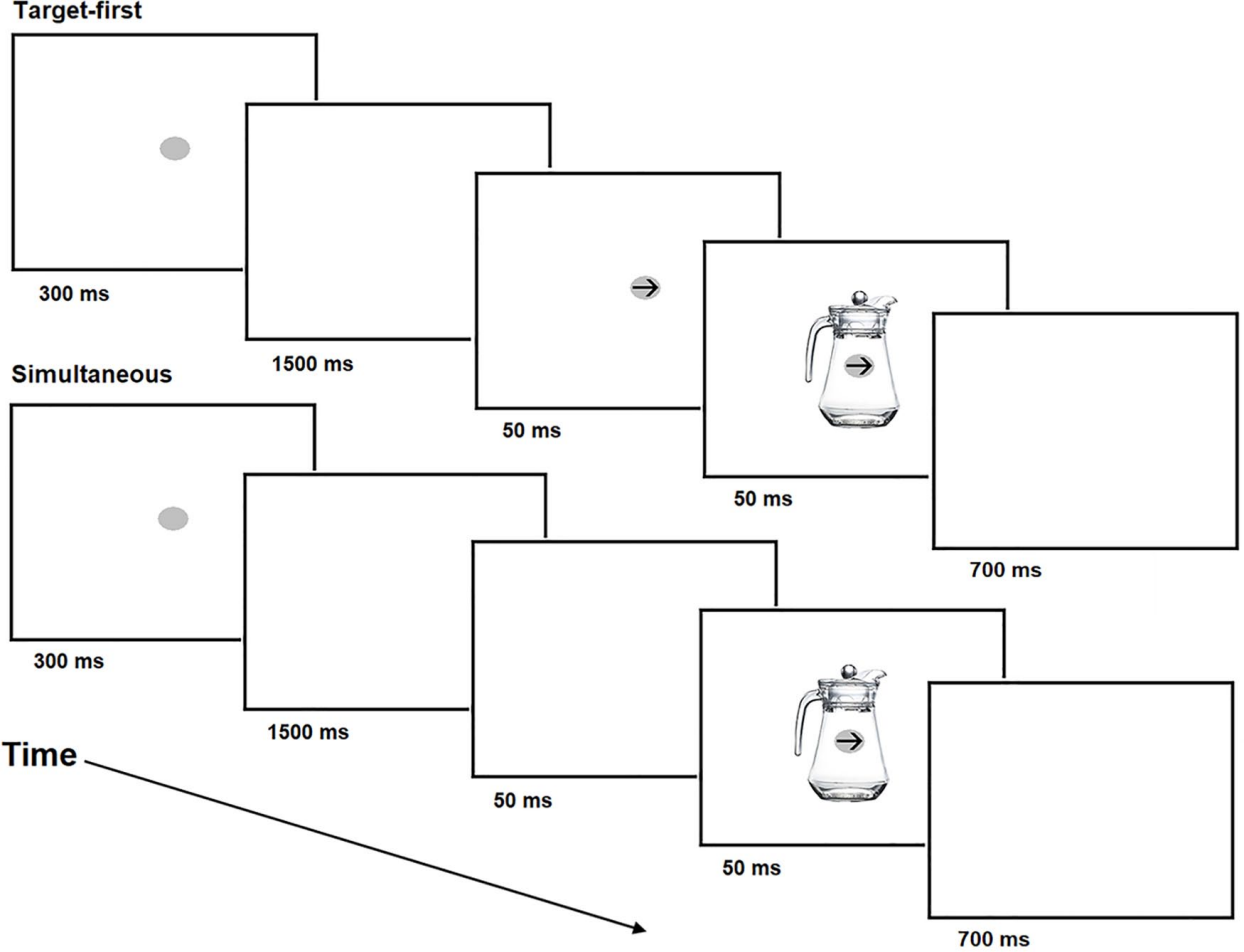
Design of Experiment 2.

**Figure 4.**
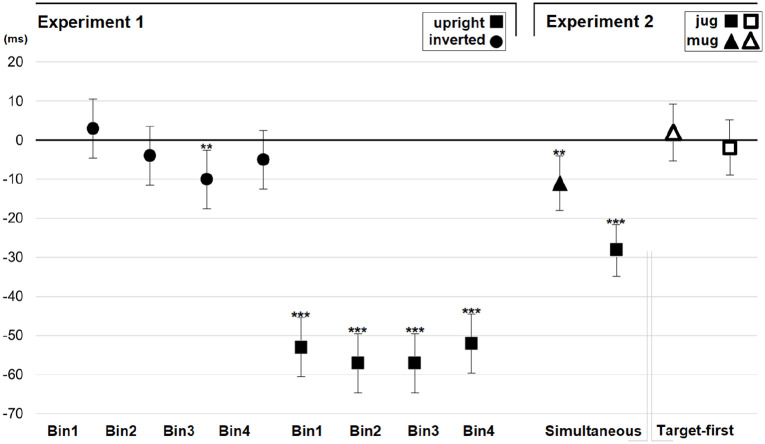
Results of Experiments 1 and 2. The compatibility effect is the difference between response times in the incompatible and compatible trials. Negative values represent faster overall responses in incompatible trials. Error bars depict the standard error of the mean. Asterisks indicate statistically significant differences (***p < .001, **p < .01, *p < .05).

To verify that the effect is based on the habitual S-R association that is acquired during lifelong practice before the study rather some other abstract S-R association acquired in the early trials of the study, the effect was explored for the first five trials of the upright block. After including these first five compatible and incompatible responses in the analysis, the analysis (BIC = 1630.34) revealed the same negative compatibility effect, *F*(1,14) = 45.31, *p* < .001; *d* = 1.4. The responses were made faster in incompatible (*M* = 361 ms) than in compatible (*M* = 424 ms) conditions.

Analysis of percentage error rates revealed a pattern of results parallel to the reaction time analysis. After estimating the best fitting error covariance structure (BIC = −2913.72), the error rates revealed significant main effects of Compatibility, *F*(1,14) = 7.93, *p* = .014, and interaction between Compatibility and Block, *F*(1,3561) = 7.42, *p* = .006. The pairwise comparisons test showed that in Block 1 (inverted), there was no significant difference between compatible (*M* = 3.0%) and incompatible (*M* = 2.0%) conditions (*p* = .331). In contrast, in Block 2 (upright) the participants made more errors in compatible (*M* = 4.8%) than in incompatible (*M* = 0.9%) conditions (*p* = .001; *d* = 1.3). That is, the participants had a tendency to execute responses with the hand that was incompatible with the handle position and withhold responding with the hand that was compatible.

#### Reaction time distributional analysis

To explore the time course of the negative compatibility effect, we carried out analysis on the distribution of reaction times with fixed within factors of Bin (1st, 2nd, 3rd, and 4th quartile) and Compatibility (Compatible, Incompatible) separately for both blocks (inverted, upright). Trials were first divided according to Compatibility and Block, and then divided separately into four bins of equal size. The analysis of the inverted block (BIC = 16503.83) revealed a significant interaction between Bin and Compatibility, *F*(3,1674) = 4.60, *p* = .003. The negative compatibility effect was only observed in Bin 3 (Comp: *M* = 389 ms, Incomp: *M* = 379 ms, *p* = .003, *d* = 0.3). The effect was missing in the rest of the bins (Bin 1: Comp: *M* = 316 ms, Incomp: *M* = 319 ms, *p* = .408; Bin 2: Comp: *M* = 357 ms, Incomp: 353 ms, *p* = .346; Bin 4: Comp: *M* = 435 ms, Incomp: *M* = 430 ms, *p* = .245). The analysis of the upright block (BIC = 16524.81) did not reveal significant interaction between Bin and Compatibility, *F*(3,1668) = 1.10, *p* = .348. A similar (*p* < .001) negative compatibility effect was observed in each bin (Bin 1: Comp: *M* = 360 ms, Incomp: *M* = 307 ms, *d* = 1.5; Bin 2: Comp: *M* = 400 ms, Incomp: 343 ms, *d* = 1.6; Bin 3: Comp: *M* = 429 ms, Incomp: *M* = 372 ms, *d* = 1.6; Bin 4: Comp: *M* = 481 ms, Incomp: *M* = 429 ms, *d* = 1.5). Figure 4 presents the outcome of this quantile analysis.

#### Responses to the oral questionnaire

(1) All participants noticed the prime object, and all participants recognised the upright jug. Six participants correctly recognised that the prime presented in the inverted block was a jug that was presented upside down. Two participants proposed that the inverted jug was some kind of blender that was presented either in an upright or inverted position. Seven participants mentioned that they did not recognise the object presented in the inverted block. (2) Two participants reported that they did not see anything changing between the presentations of the prime stimuli. Two participants reported that the location of the object changed between the trials. Eleven participants noticed that the orientation of the prime object changed between the trials. (3) Four participants suggested that in some of the trials the contrast of the prime object was dimmer than in other trials. Six participants mentioned that they were often “kind of” forced to press an incorrect response key in the upright block. They could not specify the reason for this feeling. (4) Nobody reported that they were using some strategy other than trying to focus on the target arrow and respond accordingly. (5) Nobody knew or guessed correctly the research question of the study, and nobody figured out that the handle of the prime object would in any way influence responses. (6) Nobody mentioned that they would have tried to intentionally ignore the prime object so that they could respond to the arrow accurately or to avoid responding with the hand that was compatible with the prime orientation.

### Discussion

Experiment 1 replicated the NHA effect with left–right oriented jug primes. The fact that the effect was mostly missing with inverted jug primes shows that the effect is based on functionally meaningful handle affordance information of the prime rather than some abstract featural asymmetries associated with the shifting orientation. As such, it seems that the effect is based on visuomotor processes that transmit functional affordance information to response selection via the ventro-dorsal stream. The outcomes of the study concerning automaticity of the NHA effect, which was one of the primary research questions of the study, are discussed in detail in the General discussion. However, in short, it can be stated that the findings did not support the view that the effect would be based on some voluntary strategy. In addition, the fact that the effect remained the same in each reaction time quartile suggests that, as far as this aspect is concerned, the effect might be based on somewhat the same response control mechanisms as the subliminal arrow priming, which similarly do not disappear with longer reaction times (Seiss et al., 2014). Finally, it is noteworthy that the sequence of inverted and upright blocks was not counterbalanced across the participants due to the reasons introduced in the Methods section. The inverted block was always carried out before the upright block. However, it should be emphasised that the inverted trials did not produce any (positive or negative) effect on responses. Hence, one might assume that these inverted trials should be relatively neutral regarding associating handle information with responses in the upright trials even though these upright trials are presented after the inverted block. In addition, the analysis of the first five trials of the upright block shows that the NHA effect does not develop during the study, but rather it already manifests itself in the very first trials of the block. That is, the NHA rises in the first trials of the upright block right after the zero effect of the preceding trials that present inverted objects. Moreover, the previous studies have shown that the NHA is not particularly sensitive to these kinds of sequency factors (Vainio et al., 2014). These evidences suggest that the NHA effect is not sensitive to sequential or practice influences, and does not develop during the blocks but rather appears immediately when the prime provides functional affordance information.

## Experiment 2

This experiment was structured to investigate whether the NHA effect can be preceded by a positive handle affordance effect when the delay between the prime and target is removed. This was tested by either presenting the prime and target at the same time for 50 ms, or by presenting the target 50 ms before the onset of the prime. Given that response processes can be influenced by habituated S-R associations faster than goal-directed processes (Hardwick et al., 2019), it was presumed that the handle affordance of the prime could interfere with the target-related response processes even when the prime appears 50 ms prior to the onset of the target. This study used a mug and a jug as a prime to ensure that both of these prime types produce similar priming effects.

### Method

#### Participants

Nineteen participants (14 females; 20–41 years of age; mean age = 26.6 years; 2 left-handed) had normal or corrected-to-normal vision. All persons gave their informed consent prior to their inclusion in the study. See Method section of Experiment 1 for justification of the sample size. The study was approved by the Ethical Review Board in Humanities and Social and Behavioural Sciences at the University of Helsinki.

#### Apparatus, stimuli, and procedure

The experimental arrangements and apparatus of Experiment 2 were the same as in Experiment 1. The experiment consisted of two blocks. In one block, the prime stimuli were the same picture of the upright jug that was used in Experiment 1. The stimuli of this block consisted of the same fixation point and target arrows that were used in Experiment 1. The stimuli of another block included the same mug stimuli (subtended a visual angle of 6.3° vertically and 6.5° horizontally) that were used in the original study of Vainio et al. (2011) (see Figure 1 for an illustration of this mug stimulus). In the mug block, the target arrows were the same black arrows that were used in the jug block. The order of these blocks was randomised between the participants. There was a short break between these blocks.

Each stimulus was displayed 25 times in each of the four conditions in both blocks. In total, the experiment consisted of 200 trials (25 × 2 [orientation] × 2 [hand of response] × 2 [block]). Similarly to Experiment 1, the jug and mug stimuli were presented so that their handle was pointing to the left or right. All stimuli were centralised in the display in the same way as in Experiment 1. However, contrary to Experiment 1, in Experiment 2, the target arrow either appeared and disappeared at the same time as the prime object (simultaneous condition) or the target arrow appeared in the display 50 ms before the onset of the prime and disappeared at the same time as the prime (target-first condition). The prime was always presented for 50 ms. The order of simultaneous and target-first conditions were randomised within each block. Otherwise, the task and the structure of the study was the same as in Experiment 1 (see Figure 3 for the illustration of the design).

After the second block, first (1), the participants were asked orally (oral questionnaire) whether they noticed the object that appeared in the display in addition to the arrow, and if they did notice the object, what the object in the first and second block was. The next five questions (2–6) were the same as in the oral questionnaire of Experiment 1. Finally (7), they were asked whether the arrow and the other object appeared in the display at the same time or at different times, and if they thought that they appeared at different times, which of the objects appeared first.

### Results

Reaction times were measured from the onset of the target arrow to the onset of the key press. Errors (i.e., the participant responded with a wrong hand [7.9%] or did not produce any response [0.3%]) were excluded from the reaction time analysis. In addition, reaction times faster than 200 ms (1%) were excluded from the reaction time analysis. The statistical significance of reaction time values (see Supplementary material for the data) was tested by using a random intercept model (linear mixed model) that treated Block (mug, jug), Target onset (target-first, simultaneous), and Compatibility between prime orientation and arrow direction (compatible, incompatible) as fixed within factors and Subject as random intercept. Selection of error covariance structure was based on Schwarz’s BIC. Post hoc comparisons were carried out by using the Bonferroni correction. The analysis was carried out using SPSS software package (version 25).

#### Analysis of reaction times and errors

After estimating the best fitting error covariance structure (BIC = 78203.52), the analysis of reaction times revealed significant main effects of Target onset, *F*(1,18) = 9.64, *p* = .006, and Compatibility, *F*(1,18) = 37.13, *p* < .001. Responses were made faster in the target-first (*M* = 318 ms) than in the simultaneous (*M* = 324 ms) condition, and in the incompatible (*M* = 316 ms) than in the compatible (*M* = 326 ms) conditions. The interactions between Block and Compatibility, *F*(1,7169) = 22.40, *p* < .001, Target onset and Compatibility, *F*(1,7173) = 58.49, *p* < .001, and Block, Target onset, and Compatibility, F(1,7170) = 9.83, *p* = .002, were also significant. The pairwise comparisons test showed that in the simultaneous condition of both blocks, the responses were faster in the incompatible rather than compatible conditions (Mug: Comp: *M* = 326 ms, Incomp: *M* = 316 ms, *p* = .001, *d* = 0.5; Jug: Comp: *M* = 340 ms, Incomp: *M* = 312 ms, *p* < .001, *d* = 1.3). In contrast, the compatibility effect was absent in the target-first condition of both blocks (Mug: Comp: *M* = 312 ms, Incomp: *M* = 314 ms, *p* = .407; Jug: Comp: *M* = 324 ms, Incomp: *M* = 322 ms, *p* = .495). Finally, to observe whether the negative compatibility effect was different between the mug and jug stimuli, the analysis was carried out so that simultaneous conditions were only included in the analysis. The analysis (BIC = 38761.98) revealed significant interaction between Block and Compatibility, *F*(1,3505) = 27.57, *p* < .001, *d* = 0.8, showing that although the negative compatibility effect was observed in both blocks, the effect was significantly larger with jugs than with mugs. Figure 4 presents these compatibility effects.

Similar analysis of percentage error rates revealed a pattern of results parallel to the reaction time analysis. After estimating the best fitting error covariance structure (BIC = −3450.60), the error rates revealed significant main effects of Compatibility, *F*(1,18) = 9.59, *p* = .006, and interaction between Target onset and Compatibility, *F*(1,7527) = 24.30, *p* < .001. The pairwise comparisons test showed that the compatibility effect was entirely missing in the target-first condition (Comp: *M* = 2.4%, Incomp: *M* = 2.4%, *p* = .951), while in the simultaneous condition, the participants made more errors in compatible (*M* = 7.7%) rather than incompatible (*M* = 3.3%) conditions (*p* < .001, *d* = 1.1). The interaction of Block × Target onset × Compatibility was not significant, *F*(1,7527) = 3.28, *p* = .070.

#### Responses to the oral questionnaire

(1) All participants noticed the prime objects and all participants recognised them as a jug and a mug. (2) One participant proposed that the location of the prime object shifted between the prime presentations. The rest of the participants noticed that the orientation changed between the presentations of the prime object. (3) Six participants mentioned that they were often “kind of” forced to press an incorrect response key. (4) Nobody reported that they were using some strategy other than trying to focus on the target arrow and responding accordingly. (5) Nobody knew or guessed correctly the research question of the study, and nobody figured out that the handle of the prime object would in any way influence responses. (6) Nobody mentioned that they would have tried to intentionally ignore the prime object so that they could respond to the arrow accurately or to avoid responding with the hand that was compatible with the prime orientation. (7) Two participants mentioned that the arrow might have appeared before the jug, and three participants mentioned that the jug might have appeared before the arrow. The rest of the participants mentioned that the arrow and jug were presented at the same time.

### Discussion

The NHA effect was observed with the mug and jug primes when the prime and target were presented simultaneously for 50 ms. In sharp contrast to the simultaneous condition, in the target-first condition, the effect was entirely removed with both of the prime types. This was so even though participants did not reliably report detecting this temporal asymmetry between the onsets of the prime and target. These findings reveal important aspects considering mechanisms underlying the effect. The effect does not seem to be based on the same behaviour control mechanisms of lateral inhibition that are assumed to cause the subliminal arrow priming (Bowman et al., 2006). In addition, if the model of Houghton and Tipper (1994) indeed reliably describes the mechanisms underlying the negative priming, the NHA effect is also not based on the same mechanisms as other negative priming effects because both of these models assume that the negative compatibility effect is associated with an initial response activation that is followed by response inhibition. However, the inhibitory effect associated with the handle affordance is not preceded by a brief positive compatibility effect. Rather, the inhibitory effect observed in the “simultaneous” condition arises sharply from a no-effect condition of the “target-fist.”

The results of Experiment 2 also showed that the NHA effect was significantly larger with jug primes than mug primes. There are two different possible explanations for this discrepancy. First, it has been shown that the subliminal arrow priming increases with larger primes (Lingnau & Vorberg, 2005). According to this explanation, larger primes result in stronger initial response activation than small primes that in turn require stronger response inhibition leading to larger negative compatibility effects. This view emphasises sensorimotor processing of low-level visual features in the effect. Second, the difference might be also related to processing higher-level properties of prime objects. According to this view, the discrepancy is observed because jugs provide stronger functional affordance information about the graspability of the object than mugs do. Jugs can be handled and used nearly exclusively by grasping their handle, whereas mugs are often handled and used by grasping their main body. As such, the left–right handle position of a jug is likely to provide an exceptionally strong affordance cue for the action planning processes of the hand compatible with the handle position.

## Experiment 3

Experiment 3 further explores the time course of the NHA effect by varying the interval between the prime offset and target onset (50, 100, 200, 400 ms) in a within-subjects design. If the effect complies with the time course pattern of the subliminal arrow priming, the effect should decay rapidly after offset of the prime and reverse into a positive handle affordance effect in the longest delay condition. In contrast, if the effect complies with the time course pattern of the negative priming, the negative compatibility effect should be observed in all of the delay conditions.

### Method

#### Participants

Twenty-two participants (15 females; 18–39 years of age; mean age = 26.4 years; 1 left-handed) had normal or corrected-to-normal vision. All persons gave their informed consent prior to their inclusion in the study. See Method section of Experiment 1 for justification of the sample size. The study was approved by the Ethical Review Board in Humanities and Social and Behavioural Sciences at the University of Helsinki.

#### Apparatus, stimuli, and procedure

The experimental arrangements and apparatus of Experiment 3 were the same as in Experiments 1 and 2. The stimuli consisted of the same fixation point and target arrows that were used in Experiment 1. The prime stimuli included the same mug stimuli that were used in Experiment 2. The stimuli were centralised in the display in the same way as in Experiments 1 and 2. The prime and target stimuli were presented in randomised order. The structure of the study was identical to Experiment 1 with the exception that the stimulus onset asynchrony (SOA) between the offset of the prime and onset of the target varied between 50, 100, 200, and 400 ms. The order of these SOA conditions was randomised within the experiment. Each stimulus was displayed 30 times in each of the 16 conditions. In total, the experiment consisted of 480 trials (30 × 2 [orientation] × 2 [hand of response] × 4 [SOA]).

### Results

Reaction times were measured from the onset of the target arrow to the onset of the key press. Errors (i.e., the participant responded with a wrong hand [2.8%] or did not produce any response [0.2%]) were excluded from the reaction time analysis. In addition, reaction times faster than 200 ms (3.3%) were excluded from the reaction time analysis. The statistical significance of reaction time values (see Supplementary material for the data) was tested by using a random intercept model (linear mixed model) that treated SOA (50, 100, 200, 400 ms) and Compatibility between prime orientation and arrow direction (compatible, incompatible) as fixed within factors and Subject as random intercept. Selection of error covariance structure was based on Schwarz’s BIC. Post hoc comparisons were carried out by using the Bonferroni correction. The analysis was carried out using SPSS software package (version 25).

#### Analysis of reaction times and errors

**Figure 5.**
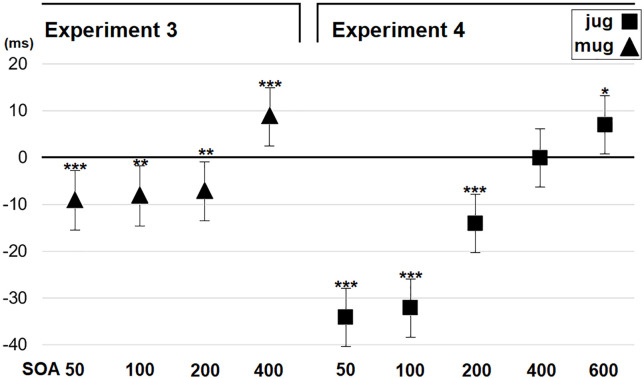
Results of Experiments 3 and 4. The compatibility effect is the difference between response times in the incompatible and compatible trials. Negative values represent faster overall responses in incompatible trials. Error bars depict the standard error of the mean. Asterisks indicate statistically significant differences (***p < .001, **p < .01, *p < .05). SOA = stimulus onset asynchrony.

After estimating the best fitting error covariance structure (BIC = 102001.75), the analysis of reaction times revealed a significant main effect of SOA, *F*(3,63) = 72.36, *p* < .001. Longer SOA conditions were associated with faster reaction times than shorter SOA conditions (SOA50: *M* = 385 ms; SOA100: *M* = 373 ms; SOA200: *M* = 363 ms; SOA400: *M* = 361 ms). More interestingly, the interaction between SOA and Compatibility was significant, *F*(3,9785) = 22.15, *p* < .001. According to the pairwise comparisons test, a significant negative compatibility effect was observed in SOAs of 50 (Comp: *M* = 389 ms, Incomp: 381 ms, *p* < .001, *d* = 0.3), 100 (Comp: *M* = 377 ms, Incomp: 370 ms, *p* = .002, *d* = 0.2), and 200 (Comp: *M* = 366 ms, Incomp: 360 ms, *p* = .007, *d* = 0.2). Importantly, the SOA condition of 400 ms produced a positive compatibility effect (Comp: *M* = 357 ms, Incomp: 365 ms, *p* < .001, *d* = 0.3). Figure 5 presents the compatibility effect in different ISI conditions.

After estimating the best fitting error covariance structure (BIC = −8224.14), the error rates revealed a significant main effect of Compatibility, *F*(1,20) = 6.09, *p* = .022, *d* = 0.3. The participants made more errors in compatible (*M* = 3.2%) than incompatible (*M* = 2.4%) conditions. The analysis did not reveal any other significant main effects or interactions.

### Discussion

The observed time course pattern of the handle affordance effect had more similarities to the subliminal arrow priming than to the negative priming. The effect decays rapidly after offset of the prime and turns into a positive compatibility effect with the longest ISI condition. That is, the negative compatibility effect was observed in the ISI conditions of 50, 100, and 200 ms, while the ISI condition of 400 ms produced a positive handle affordance effect.

## Experiment 4

The results of Experiment 2 showed that jug primes produce a significantly larger NHA effect than mug primes. As such, it is theoretically interesting to explore whether a strength of the effect modulates the time course of the effect. Hence, Experiment 4 investigates whether the negative compatibility effect triggered by the handle position of jug primes conforms to time course patterns similar to the effect triggered by the handle position of mug primes observed in Experiment 3. In addition, given that Experiment 3 provided the first attempt to demonstrate the positive reversal of the negative compatibility effect relative to the handle affordance of a non-target, for the sake of verification of this phenomenon, it is important to replicate this effect using a different prime stimulus. Moreover, in addition to including the same ISI conditions in Experiment 4 as in Experiment 3 (50, 100, 200, 400 ms), this experiment includes one more delay condition (600 ms) in the setup to investigate the time course of the positive reversal phenomenon.

### Method

#### Participants

Twenty-six participants (21 females; 20–44 years of age; mean age = 26.3 years; 3 left-handed) had normal or corrected-to-normal vision. See Method section of Experiment 1 for justification of the sample size. The number of participants was slightly increased in Experiment 4 relative to Experiment 3 because Experiment 4 included one more level of the SOA factor. All persons gave their informed consent prior to their inclusion in the study. The study was approved by the Ethical Review Board in Humanities and Social and Behavioural Sciences at the University of Helsinki.

#### Apparatus, stimuli, and procedure

The experimental arrangements, apparatus, and procedure of Experiment 4 were the same as in Experiment 3 with the exception that Experiment 5 used a new set of SOA conditions (50, 100, 200, 400, 600 ms). Each stimulus was displayed 30 times in each of the 16 conditions. In total, the experiment consisted of 600 trials (30 × 2 [orientation] × 2 [hand of response] × 5 [SOA]). The stimuli were the same left–right oriented jugs that were used in the upright block of Experiment 1.

### Results

Reaction times were measured from the onset of the target arrow to the onset of the key press. Errors (i.e., the participant responded with a wrong hand [2.7%] or did not produce any response [0.3%]) were excluded from the reaction time analysis. In addition, reaction times faster than 200 ms (0.5%) were excluded from the reaction time analysis. The statistical significance of the reaction time values (see Supplementary material for the data) was tested by using a random intercept model (linear mixed model) that treated SOA (50, 100, 200, 400, 600 ms) and Compatibility between prime orientation and arrow direction (compatible, incompatible) as fixed within factors and Subject as random intercept. Selection of error covariance structure was based on Schwarz’s BIC. Post hoc comparisons were carried out by using the Bonferroni correction. The analysis was carried out using SPSS software package (version 25).

#### Analysis of reaction times and errors

After estimating the best fitting error covariance structure (BIC = 164564.16), the analysis of reaction times revealed significant main effects of SOA, *F*(4,100) = 45.86, *p* < .001 (SOA50: *M* = 399 ms; SOA100: *M* = 389 ms; SOA200: *M* = 379 ms; SOA400: *M* = 370 ms; SOA600: *M* = 378 ms) and Compatibility, *F*(1,25) = 57.79, *p* < .001 (Comp: M = 390 ms, Incomp: M = 376 ms). More interestingly, the interaction between SOA and Compatibility was significant, *F*(4,14913) = 74.64, *p* < .001. According to the pairwise comparisons test, a significant negative compatibility effect was observed in SOAs of 50 (Comp: *M* = 416 ms, Incomp: 382 ms, *p* < .001, *d* = 1), 100 (Comp: *M* = 404 ms, Incomp: 372 ms, *p* < .001, *d* = 1), and 200 (Comp: *M* = 386 ms, Incomp: 372 ms, *p* < .001, *d* = 0.4). The SOA condition of 400 did not show any compatibility effect (*p* = .848). However, the SOA condition of 600 showed a weak positive compatibility effect (Comp: *M* = 374 ms, Incomp: *M* = 380 ms, *p* = .040, *d* = 0.2). Figure 5 presents the compatibility effect in different ISI conditions.

After estimating the best fitting error covariance structure (BIC = −13056.29), the error rates revealed significant main effects of SOA, *F*(4,100) = 4.83, *p* = .001 (SOA50: *M* = 3.4%; SOA100: *M* = 3.3%; SOA200: *M* = 2.2%; SOA400: *M* = 2.2%; SOA600: *M* = 2.1%) and Compatibility, *F*(1,25) = 12.92, *p* = .001 (Comp: *M* = 3.6%, Incomp: *M* = 1.7%). In addition, the interaction between SOA and Compatibility was significant, *F*(4,15401) = 19.88, *p* < .001. According to the pairwise comparisons test, in the SOA conditions of 50 and 100, the participants made significantly (*p* < .001) more errors in compatible rather than in incompatible conditions (SOA50: Comp: *M* = 6.0%, Incomp: *M* = 0.9%; *d* = 1.9; SOA100: Comp: *M* = 5.3%, Incomp: *M* = 1.4%, *d* = 1.5).

### Discussion

The time course of the compatibility effect observed in Experiment 4 with jug primes was observed to conform to temporal patterns observed in Experiment 3 with mug primes. In both experiments, the NHA effect lasted for 200 ms after offset of the prime. However, the positive reversal of this effect occurred later with jug primes (i.e., in the ISI of 600 ms) than mug primes (i.e., in the ISI of 400 ms). These findings, first, suggest that the time course of the inhibition phase of the NHA effect is approximately the same regardless of how strong initial inhibition is triggered by the prime affordance. Its inhibitory influence on responses is removed approximately 200 ms after offset of the prime. Second, these findings verify the reliability of the positive reversal of response inhibition triggered by handle affordance of a non-target. However, onset of the positive reversal of the effect seems to depend, to some extent, on the strength of this initial inhibition triggered by the prime affordance. The stronger the inhibition, the longer it takes for the occurrence of this positive reversal.

## General discussion

This work examined mechanisms that control habitual response associated with handle affordance of a non-target while performing goal-directed actions towards the target object. This research question was explored using the NHA effect previously observed in behavioural (Liu et al., 2016; Vainio et al., 2011, 2014) and electrophysiological (Vainio et al., 2014) patterns. The study supported the previous view that the response associated with handle affordance of a non-target is inhibited while the behavioural goal is to respond according to the target. Experiment 1 showed that this inhibition is indeed targeted at withholding the response associated with handle affordance. This is because the NHA effect was observed with the upright jug that provides functionally meaningful information about which hand would be the most suitable for grasping and manipulating the object, while the inverted jug, providing corresponding featural asymmetries linked to the handle component, did not produce the effect. In addition, the effect was observed even when, in Experiment 1, only the first five compatible and incompatible responses were included in the analysis from each participant. This supports the view that the NHA effect is based on habituated association between handle affordance and response of a particular hand acquired during lifelong practice, rather than some other S-R association between the prime and response that is acquired during the task.

One of the main aims of the study was to explore whether the NHA effect is based on automatic or voluntary response control processes. The automaticity of the effect has been previously questioned by Liu et al. (2016). However, the present study suggests that the effect is mostly based on automatic response control processes. This view was supported by the finding that, according to the oral questionnaire of Experiments 1 and 2, none of the participants figured out that the handle of the prime object would in any way influence responses. In addition, as opposed to the assumption of Liu et al. according to which the effect relies on instructing participants to intentionally ignore the prime, the effect was robustly observed throughout the study even though the appearance of the prime was not even mentioned in the instructions. The account of automaticity was also supported by the fact that the effect was observed even when none of the participants questioned in Experiments 1 and 2 reported using any intentional strategy to ignore the prime or avoid responding with the hand that was compatible with the handle position of the prime. In addition, the fact that the same effect was observed when only the first five compatible and incompatible responses were included in the analysis from each participant supports the view that the participant did not acquire any voluntary strategy during the experiment to maximise the speed and accuracy of their responses. Furthermore, it should be emphasised that the effect was observed in Experiment 2 when the prime and target were presented briefly (for 50 ms) at the same time rather than presenting the prime before the target. One might assume that in this condition it would be difficult to use the prime as some sort of strategic cue according to which the target-related responses would be adjusted (see Jaśkowski, 2008). Finally, voluntary account of the effect is poorly able to explain why the effect was observed in Experiment 2 when the prime and the target were presented simultaneously, whereas presenting the target 50 ms prior to the prime onset removed the effect even though the participants did not report detecting any temporal differences between the “target-first” and the “simultaneous” conditions. Taken together, the present study provides strong evidence to disprove the account that the NHA effect would be based on voluntary response control processes.

The present observations associated with the time course of the NHA effect suggest that the control mechanisms underlying the effect have more similarities to those underlying the subliminal arrow priming than those underlying the negative priming linked to the prime-probe tasks. That is, because similarly to the subliminal arrow priming, and differently to the negative priming, the NHA effect decayed rapidly (approximately 200 ms after prime offset). The fact that the quantile analysis of Experiment 1 revealed that the effect persists until the slowest reaction times, and that the effect reverses into a positive compatibility effect when there is 400–600 ms delay between the prime offset and target onset (Experiments 3 and 4), also suggests that the effect might be based on response control processes similar to the subliminal arrow priming. Nevertheless, even though these temporal properties suggest that the NHA effect and the subliminal priming are based on similar sensorimotor processes, there are several reasons to assume that the NHA is, in fact, based on different processes than the subliminal arrow priming.

First, one of the backbones of the view that the subliminal arrow priming is based on self-inhibition of response activation triggered by a subliminally presented prime arrow is based on the observation that the effect is associated with an activation-followed-by-inhibition pattern of the LRP (Eimer & Schlaghecken, 2003). This pattern of the LRP was not observed by Vainio et al. (2014) when they measured electrophysiological patterns of the NHA effect. Instead, the effect was associated with the emphasised motor activation supposedly needed to overcome the increased response competition under the conditions in which the target arrow is calling for the same response as the handle affordance. The view that the NHA effect is not based on an activation-followed-by-inhibition pattern was also supported by the current results of Experiment 1 that, contrary to the subliminal arrow priming (Eimer, 1999), did not reveal advantages for compatible trials in the fastest responses. In addition, this view was supported by the results of Experiment 2 that did not show any initial facilitatory phase when the delay between prime offset and target onset was removed. Rather, the inhibitory effect, which was observed when the prime and target were presented simultaneously for 50 ms, rose sharply from a no-effect condition observed when the target appeared 50 ms before the prime onset. Given that similarly to the subliminal arrow priming, the negative priming, linked to the prime-probe tasks, has also been assumed to be based on activation-followed-by-inhibition mechanisms (Houghton & Tipper, 1994), it seems that the NHA effect has to be based, at least to some extent, on some other response control mechanisms than the subliminal arrow priming or traditional negative priming. What response control mechanisms are then responsible for inhibiting responses associated with affordance of a non-target?

Vainio and Ellis (2020) proposed the possibility that the negative compatibility effect associated with the affordance of a non-target object might be based on response control mechanisms similar to those usually linked to the stop-signal and go/no-go tasks. It is generally assumed that response inhibition in these paradigms is controlled by a fronto-basal-ganglia circuit that includes, for example, specific mechanisms of the right inferior frontal gyrus, pre-supplementary motor area, and basal ganglia (Chambers et al., 2009). Although these stop-signal and go/no-go paradigms can be, in general, assumed to investigate voluntary response control processes in which participants have to intentionally stop or withhold the prepared or initiated response due to the stop signal, there is substantial evidence that the mechanisms responsible for these effects can also operate implicitly (Verbruggen et al., 2014). That is, because presenting subliminal task-relevant stop stimuli (van Gaal et al., 2008, 2009) or supraliminal task-irrelevant stop stimuli (Giesen & Rothermund, 2014; Verbruggen & Logan, 2009) slows down responses associated with these stimuli.

The interference control linked to the stop-signal paradigm is also applied in traditional response conflict paradigms of the flanker effect and Stroop effect (Friedman & Miyake, 2004), which opens the possibility that the mechanisms underlying the stop-signal effects can be utilised in different kinds of response conflict phenomena including the NHA effect. In line with this view, similarly to the NHA effect, the stop-signal effects can be assumed to be based on a “direct” inhibition of a prepared response triggered by onset of the stop signal rather than inhibition of the initial response activation associated with the onset of the stop signal (Verbruggen et al., 2014). Supporting this view of the stop-signal effect, action can be inhibited by a habitual stop stimulus a mere 100 ms after the onset of this stimulus (Chiu et al., 2012). The observation that successful response inhibition in both of these effects―the NHA effect and the stop-signal effect―is associated with increased brain activation associated with processes required to overcome the increased response conflict (Aron et al., 2007; Vainio et al., 2014) also suggests that the NHA effect is indeed based on response control mechanisms similar to the stop-signal effects. Moreover, it is important to stress that the excitatory phase that follows response inhibition, observed in Experiments 3 and 4, can be assumed to occur also in relation to mechanisms that control responses in the stop-signal paradigm (Sano et al., 2013). Finally, there is evidence that response inhibition in the stop-signal paradigm can become a learned reflex, becoming automatised with practice, which is easily triggered by habitualized stop stimuli (Verbruggen et al., 2014). In the context of handle affordance, this could be taken to mean that grasping the handle of the objects becomes a habitual response through continual practice, whereas when this S-R association is in turn linked to a non-target object, inhibition of response associated with the handle becomes a learned reflex.

In conclusion, the study reveals some fundamental aspects of mechanisms underlying inhibitory control of habitual responses associated with a non-target object while performing goal-driven responses. These control processes operate automatically, instead of voluntarily, relative to habitual responses associated with a non-target object. In addition, these response control processes are likely to share the mechanisms with those that are responsible for inhibiting responses in the stop-signal paradigms, enabling “direct” inhibition of the habituated response (i.e., response inhibition in the absence of preceding initial response excitation). Assumable, as a complementary element of the mechanism that programmes habitual responses, individuals are habitually conditioned to inhibit responses that are associated with affordances of a non-target. If relevant knowledge regarding functions of the parieto-frontal network is applied to the understanding of these response control processes underlying the NHA effect, one might assume that affordance of a viewed object is rapidly and implicitly identified in the parietal mechanisms of the ventro-dorsal stream. While performing goal-directed actions, detection of this affordance cue associated with a non-target automatically (i.e., as a learned reflex) triggers inhibition of a matching action representation.

## Research Data

sj-xlsx-1-qjp-10.1177_1747021820971921 – Research Data for Automatic inhibition of habitual response associated with a non-target object while performing goal-directed actions Click here for additional data file. Research Data, sj-xlsx-1-qjp-10.1177_1747021820971921 for Automatic inhibition of habitual response associated with a non-target object while performing goal-directed actions by Lari Vainio in Quarterly Journal of Experimental Psychology https://creativecommons.org/licenses/by/4.0/ This article is distributed under the terms of the Creative Commons Attribution 4.0 License ( http://www.creativecommons.org/licenses/by/4.0/) which permits any use, reproduction and distribution of the work without further permission provided the original work is attributed as specified on the SAGE and Open Access pages ( https://us.sagepub.com/en-us/nam/open-access-at-sage).
